# Management of osteoporosis and associated quality of life in post menopausal women

**DOI:** 10.1186/1471-2474-12-7

**Published:** 2011-01-12

**Authors:** Bernard Cortet, Francis Blotman, Françoise Debiais, Dominique Huas, Florence Mercier, Chantal Rousseaux, Véronique Berger, Anne-Françoise Gaudin, François-Emery Cotté

**Affiliations:** 1 Rheumatology Department, Hôpital Roger Salengro, Lille, France; 2 Rheumatology Department, Montpellier University Hospital, Montpellier, France; 3 Rheumatology Department, Poitiers University Hospital, Poitiers, France; 4 General Practice Department, UFR Tours, Tours, France; 5 STAT-Process, Port-Mort, France; 6 Nukléus, Paris, France; 7 Laboratoire GlaxoSmithKline, Marly-le-Roi, France

## Abstract

**Background:**

The study aimed to describe the characteristics of women treated for recently-diagnosed osteoporosis, to identify variables associated with different treatment regimens and to assess impact on quality of life.

**Methods:**

This is an observational, cross-sectional pharmacoepidemiological study performed in France. A random sample of 684 general practitioners, gynaecologists and rheumatologists included the first three post-menopausal osteoporotic women consulting in the previous six months on the basis of densitometry or fracture. Data on osteoporosis, fracture risk factors, treatments and comorbidities was collected with a physician questionnaire. Data on quality of life was collected using the SF-12.

**Results:**

Data were analysed for 1,306 patients, of whom 1,117 (85.5%) had been evaluated by densitometry within the previous six months and 554 (42.4%) had experienced a fracture, most frequently of the spine or wrist within the previous six months. Osteoporotic fracture risk factors were reported in 1,028 women (78.7%). 746 women (57.1%) were currently receiving treatment, most frequently weekly or monthly bisphosphonates. Five variables were associated with prescription choice: age (*p *< 0.0001), physician speciality (*p *< 0.0001), previous fracture history (*p *= 0.0002), ongoing treatment at the time of consultation (*p *= 0.0091) and paraclinical investigations performed in the previous six months (*p *= 0.0060). SF-12 scores were lower in women complaining of pain, with recent fractures and with spine or hip fractures and in women consulting rheumatologists.

**Conclusions:**

A high proportion of women diagnosed with osteoporosis had been evaluated by densitometry, in agreement with national guidelines. Treatment choice varied between physician groups.

## Background

Osteoporosis is the most frequent pathological cause of skeletal weakening, characterised by a concomitant reduction in bone mass and in bone quality, leading to an increased risk of fracture. Osteoporosis is most common in post-menopausal women, due to loss of trophic support for bone tissue from sex hormones, and is defined in this population as a bone mineral density (BMD) more than 2.5 standard deviations below the average value in young women. It has been estimated that around 30% of all post-menopausal women fulfil this definition [1]. The prevalence of osteoporosis, as well as the associated fracture risk, both rise with age [1].

Although the risk of osteoporotic fracture can however be reduced by timely diagnosis of bone mineral loss using densitometry and implementation of a specific antiresorptive or anabolic treatment [2], osteoporosis remains underdiagnosed and undertreated [3-6]. Given the importance of osteoporotic fractures to public health, national health services have been recommending more widespread availability of bone densitometry to identify those women most at risk for osteoporotic fractures. For example, the National Osteoporosis Foundation in the United States has been recommending routine bone densitometry for all women over 65 since 1999 [7].

In 2006, the national health authorities in France (Haute Autorité de Santé, HAS) published guidelines for the use of densitometry which covered the technology to be used, conditions and frequency of use and the populations to whom it should be offered [8]. These recommended that densitometry should be offered to post-menopausal women presenting specific risk factors for osteoporosis or osteoporotic fracture. These include previous fractures, corticosteroid treatment, certain endocrine diseases, hip fracture in a first degree relative, low body mass and early menopause. In parallel, the HAS introduced for the first time systematic reimbursement for densitometry in such women as well as for specific anti-osteoporosis treatments for women in whom a pathologically low bone mass density was observed. Before the introduction of these guidelines, a number of studies performed in France indicated that up to one half of women receiving a diagnosis of osteoporosis only did so after the occurrence of a first fracture [3,9,10]. In addition, a large primary care survey performed in 2005 [10] reported that less than forty percent of postmenopausal women who had not experienced a fracture had undergone densitometry.

It is therefore timely and pertinent to evaluate the impact of these new French guidelines on diagnosis and treatment of post-menopausal osteoporosis in France. For this reason, we have carried out a pharmacoepidemiological survey of osteoporosis and its treatment in a primary and secondary care environment in France. The primary objective of the study was to describe the characteristics of women receiving treatment for osteoporosis diagnosed in the previous six months. Secondary objectives were to identify variables potentially associated with different treatment regimens, to assess impact on quality of life, to compare patient and treatment characteristics between prescriber groups (general practitioners, rheumatologists and gynaecologists) and to evaluate treatment compliance and patient satisfaction with, their anti-osteoporosis treatment. Data on compliance and patient satisfaction from this study have been published previously [11]. The current article presents data on the extent of use of densitometry, on the prescription of specific osteoporosis treatments and on quality of life.

## Methods

This was an observational, cross-sectional pharmacoepidemiological study performed in France. The study was conducted in the context of primary and specialist care and collected data from physicians. Three medical specialities participated in the study, namely general practice, rheumatology and gynaecology. These specialties are responsible for essentially all diagnosis of osteoporosis in France although, following diagnosis, most patients are followed in general practice [9]. Physicians were recruited into the study between November 2007 and April 2008. Each physician recruited patients into the study over a three-month period. The last patient was entered into the database in July 2008.

### Participating physicians

General practitioners (GPs), gynaecologists and rheumatologists participated in the study. These were selected at random from an exhaustive national physician database made available by an independent medical information company (CEGEDIM: *CEntre de GEstion, de Documentation, d'Informatique et de Marketing*, Boulogne-Billancourt, France), which is widely used in epidemiological studies in France. An initial list of 10,000 physicians was constituted using a sampling method stratified by region. This list respected a ratio of three GPs to one rheumatologist and to one gynaecologist. The relative weight of the different medical specialities was chosen to reflect their role in the overall management of osteoporosis in France, determined from a recent general population study (INSTANT study) of osteoporosis in France [9]. The planned number of participating physicians was 650, based on each participant recruiting three patients to reach a total sample of 1950 patients (see below).

### Subjects

Participating investigators included consecutively into a patient registry all women who came for consultation during the three-month recruitment period and who had undergone bone densitometry in the previous six months or who had experienced a fracture not related to trauma or cancer in the previous six months. In order to avoid centre effects, the number of women that each investigator could include in the registry was limited to the first ten. Women entered into the patient registry constituted the registry population.

For each woman included in the patient registry, three eligibility criteria were ascertained. These were (i) post-menopausal status, (ii) a diagnosis of osteoporosis made in the previous six months on the basis of either low bone density (vertebral T score or total hip T score at least 2.5 standard deviations lower than the mean value in young women in France) determined by densitometry or the occurrence of an osteoporotic fracture, and (iii) an anti-osteoporosis treatment initiated in the previous six months or planned at the time of the consultation. Women who had participated in any study likely to have influenced their treatment were excluded, as were women who could not read or write. The first three women in the patient registry who fulfilled all these criteria were entered into the study and constituted the study analysis population. All women in the study analysis population were provided with a patient questionnaire to complete and those who returned an exploitable questionnaire constituted the patient questionnaire population.

### Data collection

Participating physicians provided general professional information and specific information on osteoporosis management. For each patient included in the registry, the physician noted the age of the patient, the age at menopause (if this had occurred), the age at which osteoporosis was diagnosed (if this was the case), information on densitometry, fractures, fracture risk factors and any current or planned osteoporosis treatments. The risk factors considered included osteoporosis risk factors listed in current French guidelines [8] and general fracture risk factors. The following were considered: previous fracture history (after the age of 40 without major trauma), family history of hip fracture without major trauma or osteoporotic fracture, menopause before the age of 40, BMI < 19 kg/m^2^, alcohol consumption >14 glasses per week, smoking, long term corticosteroid treatment (>3 months), bed-bound> 3 months, breast cancer, medication that may cause falls (*eg *benzodiazepines, antiepileptic drugs), endocrine disease, rheumatoid arthritis, recent decrease in visual acuity, poor general health (more than three comorbidities).

For the patients in the study analysis population, each participating physician completed a medical questionnaire. This included items on height, weight, exercise, fracture history, osteoporosis management, comorbidities, and comedication. In addition, the physician provided these patients with a questionnaire to complete. This collected data on sociodemographic features, lifestyle, attitudes and knowledge concerning osteoporosis and its treatment, treatment compliance, treatment satisfaction and quality of life. Treatment compliance was evaluated with the Morisky-Green questionnaire [12] and quality of life with the SF-12 health profile measure [13]. Both were used in their validated French translations.

### Statistical analysis

*A priori *power calculations were performed in order to determine the target sample size. In order to determine treatment frequencies of 50% with a precision of 2.5% and an α risk of 0.05, it would be necessary to include 1,536 patients. A non-participation rate of 25% was anticipated, leading to a total sample size of 1,950 patients.

The analysis was restricted to those patients for whom exploitable data was available both for the patient registry and for the medical questionnaire. Statistical comparisons were performed using the χ^2 ^test or Fisher's exact test for categorical variables and analysis of variance or the Wilcoxon test for quantitative variables. All tests were two-tailed. A probability threshold of 0.05 was taken as statistically significant. Determinants of treatment were evaluated by multivariate logistic regression analysis using a rising stepwise procedure with a cut-off probability threshold of 0.1 at each step, in which all variables whose frequencies differed between treatment groups at a probability level of 0.2 in univariate analysis were initially entered. A final multivariate model was generated in which only variables retained in the stepwise model were entered in order to generate odds ratios for the association with treatment group. Data were analysed using SAS^® ^software, Version 8.2 (SAS, Cary, USA) on Windows.

### Ethics

The survey protocol was submitted for evaluation to the CCTIRS (National Ethics Advisory Board). The board considered that participation of patients in the study would not affect their medical care, and therefore that it was not necessary to obtain formal Ethics Committee approval nor to collect signed informed consent from each patient. The only requirement stipulated was that formal information on the goals and methods of the study be provided for each patient. The study protocol was submitted to the Commission Nationale de L'informatique et des Libertés (CNIL), responsible for overseeing data privacy in France.

## Results

### Participating physicians

Overall, 684 physicians participated in the study and included patients in the registry. These included 420 GPs, 154 rheumatologists and 110 gynaecologists. The sociodemographic characteristics of participating physicians were compared to those of all physicians in France. GPs were older than the national average (51.3 years versus 48.7 years). Male GPs (84.0% versus 70.2%) and gynaecologists (31.5% versus 10.9%) and female rheumatologists (39.9% versus 32.0%) were over-represented compared to national statistics. For GPs (9.6% versus 18.0%) and rheumatologists (14.3% versus 23.9%), physicians practicing in Paris were under-represented.

### Subjects

Overall, 5,336 women fulfilled one of the two entry criteria for the registry (bone densitometry or a probable osteoporotic fracture in the last six months) and were entered into the patient registry. Of these registry patients, 2,155 (40.4%) fulfilled all three criteria for the main study (post-menopausal status, osteoporosis diagnosis and osteoporosis treatment). Two hundred women fulfilled the first two criteria but no treatment was prescribed or envisaged by the physician.

**Figure 1:**
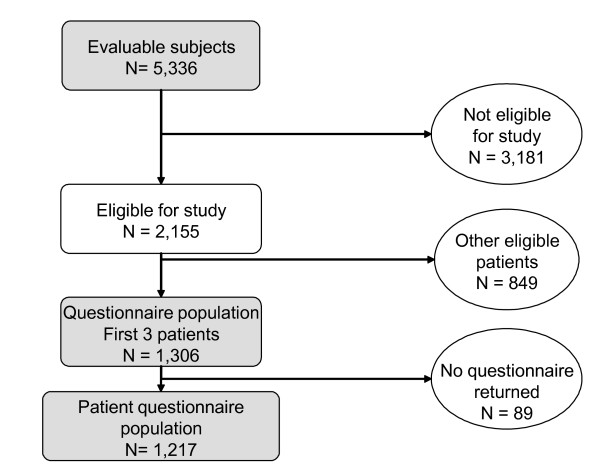
**Patient flow through the study**. Shaded boxes indicate the patient groups analysed.

The first three patients of this group consulting each participating physician were entered into the main study and provided with a questionnaire to complete. This group of 1,306 women constituted the study analysis population. The remaining 849 eligible patients were supernumerary to requirements for the study analysis population. Completed patient questionnaires were received from 1,217 (93.2%) women who constituted the patient questionnaire population. The flow of patients through the study is illustrated in Figure 1.

The women included in the study analysis population were compared with the other eligible women who did not fall within the first three patients included in the registry. Several differences were observed. Women in the study analysis population were significantly but marginally younger (66.2 ± 8.9 years *versus *67.3 ± 9.7 years; *p *= 0.007, Kruskall-Wallis test), had been more frequently evaluated by densitometry (85.5% *versus *74.3%; *p *≤ 0.001, χ^2 ^test), reported less fractures (42.4% *versus *58.7%; *p *≤ 0.001, χ^2 ^test) and were more frequently already treated at the time of the consultation (60.1% *versus *40.1%; *p *≤ 0.001, χ^2 ^test). Considering all women eligible for the study in the registry population, 1,744 (81.3%) had undergone densitometry in the previous six months and 1,052 (49.0%) had experienced a fracture.

Since this unexpected difference suggested that patients diagnosed by densitometry were being entered into the study more often than patients diagnosed due to a fracture, a *post hoc *analysis was performed to compare these two groups of patients (Table 1). Women having undergone densitometry were younger than those having experienced a fracture in the previous six months and were more recently postmenopausal. They presented less fracture risk factors and were more frequently prescribed SERMs. Women who had both undergone densitometry and experienced a fracture in the previous six months were generally speaking similar to the women who had experienced a fracture but not undergone densitometry.

**Table 1:** Osteoporosis features in the study analysis population as a function of diagnostic criterion for osteoporosis

	Low BMD only N = 752	Fracture only N = 189	Both low BMD and fracture N = 365	*p*	Total N = 1,306
**Age at inclusion** (mean ± SD; years)	*N = 750* 64.3 ± 8.4	*N = 189* 70.6 ± 9.6	*N = 365* 67.7 ± 8.671	≤ 0.001	*N = 1304* 66.2 ± 8.9
**Age at menopause** (mean ± SD; years)	*N = 745* 49.8 ± 4.0	*N = 187* 50.0 ± 3.6	*N = 360* 49.3 ± 4.3	0.218	*N = 1290* 49.7 ± 4.0
**Time since menopause** (mean ± SD; years)	*N = 745* 14.5 ± 8.7	*N = 187* 20.6 ± 9.5	*N = 360* 18.3 ± 9.9	≤ 0.001	*N = 1290* 16.5 ± 9.4
**At least one risk factor for osteoporosis**	564 (75.0%)	156 (82.5%)	308 (84.4%)	≤ 0.001	1,028 (78.7%)
**Osteoporosis treatment ongoing at time of consultation**	427 (56.8%)	118 (62.4%)	240 (65.8%)	0.016	785 (60.1%)
**Nature of ongoing treatment**	*N = 407*	*N = 111*	*N = 228*		*N = 746*
Bisphosphonates	295 (72.5%)	084 (75.7%)	164 (71.9%)	0.895	543 (72.8%)
Selective estrogen receptor modulator	53 (13.0%)	7 (6.3%)	16 (7.0%)	0.023	76 (10.2%)
Strontium ranelate	54 (13.3%)	18 (16.2%)	47 (20.6%)	0.062	119 (16.0%)
Other	14 (3.5%)	6 (5.4%)	6 (2.7%)	0.056	26 (3.5%)
**Calcium or vitamin D supplementation**	*N = 597* 609 (82.3%)	*N = 153* 161 (86.6%)	*N = 324* 316 (87.8%)	0.008	*N = 1064* 1086 (84.5%)

Data are presented as mean ± SD for quantitative variables and as numbers of women (%) for categorical variables. Between group differences were determined with the Kruskal-Wallis test or Fisher's exact test, as appropriate.

In the study analysis population, 1,117 women (85.5%) had undergone densitometry and 554 (42.4%) had experienced a fracture in the previous six months. Of these, 365 (27.9% of the study analysis population) had both undergone densitometry and experienced a fracture in the previous six months. In addition, it should be noted that 57 women who had undergone bone densitometry had experienced a fracture prior to the six month cut-off for the time horizon specified in the eligibility criteria. The total number of women in the study analysis population with a fracture history was thus 611 (46.8%). We also evaluated the reasons for assignment of a diagnosis of osteoporosis. In the study analysis population, 815 women (62.4%) had received their diagnosis on the basis of a low bone density only, 269 (20.6%) on the basis of a fracture only, and 222 (17.0%) on the basis of both criteria. Densitometry was thus taken into account for the diagnosis of 1037 women overall (79.4%).

In the study analysis population, 810 women had been included by GPs (62.0%), 317 by rheumatologists (24.3%) and 179 by gynaecologists (13.7%). The osteoporosis characteristics of these patients according to medical speciality are presented in Table 2. For the 1,037 women who had undergone densitometry, the mean T score was -2.19 ± 1.14 at the hip and -2.38 ± 1.31 in the spine. Of these women, 890 (91.5%) has a T score ≤ 2.5 standard deviations below normative values consistent with a diagnosis of osteoporosis. Women consulting a specialist were more likely to have undergone densitometry than women consulting GPs. For the 611 women who had experienced a fracture, these were most frequently vertebral (in 44.9% of women) or wrist (in 42.0%) fractures. The mean number of previous fractures in this group was 1.47 ± 0.89. Fractures were reported more frequently in women consulting a GP or a rheumatologist than in those consulting a gynaecologist. Risk factors for osteoporotic fractures were most frequent in women consulting a rheumatologist and least frequent in those consulting a gynaecologist.

**Table 2:** Osteoporosis features in the study analysis population as a function of treating physician

	GPs N = 810	Rheumatologists N = 317	Gynaecologists N = 179	*p*	Total N = 1,306
**Age **(mean ± SD; years)	*N = 809* 66.4 ± 8.6	*N = 317* 68.6 ± 9.5	*N = 178* 60.7 ± 7.0	≤ 0.001	*N = 1,305* 66.2 ± 9.2
**Densitometry in the last 6 months**	*N = 806* 651 (80.8%)	*N = 316* 296 (93.7%)	*N = 179* 170 (95.0%)	≤ 0.001	*N = 1301* 1,117 (85.9%)
**T score **(mean ± SD)					
Hip	*N = 574* -2.15 ± 1.25	*N = 290* -2.28 ± 1.00	*N = 166* -2.15 ± 0.96	0.125	*N = 1,030* -2.19 ± 1.14
Spine	*N = 573* -2.29 ± 1.48	*N = 287* -2.50 ± 1.09	*N = 167* -2.49 ± 0.97	0.167	*N = 1,027* -2.38 ± 1.31
**T score by class**	*N = 577*	*N = 254*	*N = 142*		*N = 973*
Normal BMD (T score < -1)	0 (0.0%)	2 (0.8%)	0 (0.0%)	0.192	2 (0.2%)
Osteopenia (-2.5 < T score < -1)	53 (9.2%)	17 (6.7%)	11 (7.8%)		81 (8.3%)
Osteoporosis (T score ≤ -2.5)	524 (90.8%)	235 (92.5%)	131 (92.3%)		890 (91.5%)
**Osteoporotic fractures**	N = 777 407 (52.4%)	N = 311 177 (56.9%)	N = 162 27 (16.7%)	≤ 0.001	*N = 1,250* 611 (48.9%)
**Number of osteoporotic fractures **(mean ± SD)	*N = 386* 1.38 ± 0.70	*N = 169* 1.68 ± 1.18	*N = 27* 1.33 ± 0.88	0.021	*N = 582* 1.47 ± 0.89
**Fracture site**	*N = 376*	*N = 148*	*N = 26*		*N = 550*
Spine	162 (43.1%)	77 (52.0%)	8 (30.8%)	0.060	247 (44.9%)
Wrist	173 (46.0%)	43 (29.1%)	15 (57.7%)	≤ 0.001	231 (42.0%)
Upper arm	18 (04.8%)	8 (05.4%)	1 (03.9%)	0.932	27 (4.9%)
Hip	42 (11.2%)	10 (06.8%)	0 (0.0%)	0.081	52 (9.5%)
Other	35 (09.3%)	27 (18.2%)	7 (26.9%)	0.002	69 (12.6%)
**Patients with at least one risk factor for osteoporosis**	639 (78.9%)	260 (82.0%)	129 (72.1%)	0.037	1028 (78.7%)
**Diagnosis of osteoporosis**	N = 810	N = 317	N = 179	< 0.001	222 (17.0%)
Low BMD + osteoporotic fracture	126 (15.6%)	087 (27.4%)	009 (05.0%)		222 (17.0%)
Low BMD only	495 (61.1%)	160 (50.5%)	160 (89.4%)		815 (62.4%)
Osteoporotic fracture only	189 (23.3%)	070 (22.1%)	010 (05.6%)		269 (20.6%)

Between group differences were determined with the Kruskal-Wallis test or Fisher's exact test, as appropriate.

### Osteoporosis treatments

Around half of women were currently receiving treatment (N = 746; 57.1%). The most frequently prescribed treatments already initiated at the time of treatment were weekly or monthly bisphosphonates (Table 3). Daily bisphosphonates, hormone replacement therapy (HRT) and parathyroid hormone analogues (PTH) were prescribed to less than five percent of women. The treatments listed in this Table cover all osteoporosis treatments approved in France at the time of the study. For this reason, HRT (n = 26) and PTH (n = 11) treatment were combined in an "Other treatments" group. Treatments had been prescribed for approximately three months at the time of the consultation (data not shown). General practitioners renewed the prescriptions of around two-thirds of their patients (64.9%), whereas this was the case for less than half of patients consulting rheumatologists (47.3%) and gynaecologists (39.1%). Gynaecologists prescribed bisphosphonates less frequently than the other physician groups, and prescribed hormone replacement therapy and selective oestrogen receptor modulators (SERMs) more frequently (Table 3). Dietary supplementation with calcium and vitamin D was prescribed to the large majority of women by all physician groups.

**Table 3:** Treatments in the study analysis population as a function of treating physician

	GPs N = 810	Rheumatologists N = 317	Gynaecologists N = 179	*p*	Total N = 1306
**ON GOING TREATMENT**	526 (64.9%)	150 (47.3%)	70 (39.1%)	≤ 0.001	746 (57.1%)
Bisphosphonates	412 (78.3%)	98 (65.3%)	33 (47.1%)	≤ 0.001	543 (72.8%)
*Daily*	14 (3.5%)	2 (2.1%)	1 (3.2%)	NS	17 (3.2%)
*Weekly*	227 (56.9%)	60 (63.8%)	18 (58.1%)		305 (58.2%)
*Monthly*	158 (39.6%)	32 (34.0%)	12 (38.7%)		202 (38.6%)
SERM	47 (8.9%)	14 (9.3%)	15 (21.4%)	0.009	76 (10.2%)
Strontium ranelate	68 (12.9%)	34 (22.7%)	17 (24.3%)	0.003	119 (16.0%)
Other	10 (1.9%)	4 (2.7%)	12 (17.1%)	≤ 0.001	26 (3.5%)
**SUPPLEMENTATION**	671 (82.8%)	269 (84.9%)	146 (81.6%)	NS	1086 (83.2%)
**Supplementation Type**	*N = 655*	*N = 268*	*N = 141*		*N = 1064*
Calcium	33 (5.0%)	7 (2.6%)	3 (2.1%)	0.073	43 (4.0%)
Vitamin D	27 (4.1%)	20 (7.5%)	9 (6.4%)		56 (5.3%)
Calcium + Vitamin D	595 (90.8%)	241 (89.9%)	129 (91.5%)		965 (90.7%)

For the individual osteoporosis treatments, only treatments ongoing at the time of the consultation are considered. Percentages for individual treatments are calculated with respect to the total number of patients with an ongoing treatment. Percentages for bisphosphonate treatment regimens are calculated with respect to the total number of patients taking a bisphosphonate. Percentages for supplementation type are calculated with respect to the total number of patients taking supplementation. SERM: selective oestrogen receptor modulators. NS: not significant. Between group differences were determined with the Kruskal-Wallis test.

### Variables associated with treatment choice

In a first step, all variables recorded in the registry and medical questionnaire were evaluated for their potential association with the class of treatment prescribed using univariate analysis. Given the small number of subjects concerned, the 'Other treatments' and 'Daily Bisphosphonates' groups were not taken into account in this analysis. In the univariate analysis, significant associations were observed for a large number of variables at the prespecified probability threshold of 0.2. Those specifically related to osteoporosis are presented in Table 4. Most inter-group differences corresponded to a difference between SERMs on the one hand and bisphosphonates and strontium ranelate on the other. Selective oestrogen receptor modulators were more likely to be prescribed to younger women, those without objectively demonstrated osteoporosis by densitometry and in those without previous fractures. In addition, a diagnosis of osteoporosis was more frequently already assigned (p = 0.038) at the time of consultation to patients prescribed weekly bisphosphonates (71.8%) and strontium ranelate (72.5%) than to those prescribed monthly bisphosphonates (65.6%) or SERMs (60.4%).

**Table 4:** Treatment choice - univariate analysis

		BP Weekly N = 462	BP Monthly N = 388	Strontium Ranelate N = 182	SERM N = 115	*p*	Total N = 1180
**Physician speciality**	Rheumatologists	122 (26.4%)	086 (22.2%)	053 (29.1%)	022 (19.1%)	≤ 0.001	283 (24.7%)
	Gynaecologists	044 (09.5%)	038 (09.8%)	047 (25.8%)	036 (31.3%)		165 (14.4%)
	GPs	296 (64.1%)	264 (68.0%)	082 (45.1%)	057 (49.6%)		699 (60.9%)
**Age**	Mean ± SD	67.3 ± 9.2	66.9 ± 8.1	65.3 ± 9.3	60.00 ± 6.0	≤ 0.001	66.1 ± 8.9
	*Missing data*	*1*	*0*	*1*	*0*		*2*
**Time since menopause **(years)	Mean ± SD	17.3 ± 9.6	17.2 ± 9.0	15.7 ± 9.8	10.2 ± 6.3	≤ 0.001	16.3 ± 9.4
	*Missing data*	*5*	*3*	*1*	*3*		12
**Type of osteoporosis diagnosis**	Low BMD + osteoporotic fracture	097 (21.0%)	060 (15.5%)	031 (17.0%)	008 (07.0%)	≤ 0.001	196 (17.1%)
	Low BMD without osteoporotic fracture	271 (58.7%)	259 (66.8%)	106 (58.2%)	097 (84.4%)		733 (63.9%)
	Osteoporotic fracture without low BMD	094 (20.4%)	069 (17.8%)	045 (24.7%)	010 (08.7%)		218 (19.0%)
**BMD**	N (%)	391 (84.8%)	331 (86.0%)	159 (87.9%)	108 (93.9%)	0.057	989 (86.6%)
	*Missing data*	*1*	*3*	*1*	*0*		*5*
**Mean Hip T-score**	Mean ± SD	-2.24 ± 1.12	-2.34 ± 0.79	-2.33 ± 1.01	-1.81 ± 1.40	0.004	-2.24 ± 1.05
	*Missing data*	*1*	*1*	*1*	*0*		*3*
**T-Score by class**	Normal BMD (Tscore>-1)	1 (0.3%)	0 (0.0%)	1 (0.7%)	000 (00.0%)	0.074	002 (00.2%)
	Osteopenia (-2.5 < Tscore <-1)	30 (8.7%)	16 (05.5%)	7 (5.0%)	012 (13.0%)		065 (07.5%)
	Osteoporosis (Tscore≤-2.5)	313 (91.0%)	275 (94.5%)	131 (94.2%)	080 (87.0%)		799 (92.3%)
	*Missing data*	*47*	*40*	*20*	*16*		*127*
**Osteoporotic fractures**		230 (51.6%)	169 (46.3%)	95 (54.0%)	27 (25.0%)	≤ 0.001	521 (47.6%)
	*Missing data*	*16*	*23*	*6*	*7*		*52*
Fractures in the previous 6 months		205 (44.7%)	151 (39.3%)	89 (49.2%)	023 (20.0%)	≤ 0.001	468 (41.1%)
	Missing data	*3*	*4*	*1*	*0*		*8*
Number of fractures in the previous 6 months		1.28 ± 0.62	1.47 ± 0.88	1.40 ± 0.73	1.33 ± 0.58	0.195	1.37 ± 0.74
	*Missing data*	24	17	6	2		49
**Long-term corticosteroid treatment (> 3 months)**		47 (10.2%)	36 (9.3%)	11 (6.0%)	5 (4.4%)	0.122	99 (8.6%)
**Ongoing treatment at the time of consultation**		301 (65.2%)	216 (55.7%)	114 (62.6%)	066 (57.4%)	0.032	697 (60.8%)
**Paraclinical investigations**		433 (93.7%)	374 (96.6%)	175 (96.2%)	105 (91.3%)	0.057	1087 (94.9%

These variables, together with all others whose distribution varied between treatment classes at the prespecified probability threshold (body mass index, comorbid, atherosclerosis or arterial hypertension, history of myocardial infarction, physical activity, presence of a comorbidity, comedication for chronic diseases, patient concern about osteoporosis; importance of osteoporosis management for the physician) were entered into the stepwise multivariate regression analysis. However, T-scores determined in the densitometry evaluations were not included as this information was missing for around one-quarter of the subjects. Only five variables were retained, namely age (*p *< 0.0001), physician speciality (*p *< 0.0001), previous fracture history (*p *= 0.0002), ongoing treatment at the time of consultation (*p *= 0.0091) and paraclinical investigations performed in the previous six months (*p *= 0.0060).

In a final step, the five variables retained in the stepwise analysis were entered into a final multiple logistic regression model for estimation of odds ratios. This analysis was restricted to the 1,092 patients for whom no data was missing. The derived odds ratios are presented in Table 5. Weekly bisphosphonates were more likely to be prescribed to older women than were the other therapeutic classes. Strontium ranelate was more likely to be prescribed to women with a previous fracture history. Treatment with monthly bisphosphonates was more likely to be prescribed to women who had undergone paraclinical investigations and also less likely to be already underway at the time of the consultation than in women receiving weekly bisphosphonate treatment. Compared to rheumatologists, gynaecologists were more likely to prescribe SERMs, and GPs less likely to prescribe strontium ranelate and more likely to prescribe monthly bisphosphonates.

**Table 5:** Odds ratios variables independently associated with treatment choice identified by multivariate regression analysis

Variable	Treatment modality (Reference: Weekly BP)	Physician speciality	Odds Ratio	P
Age	Monthly bisphosphonates		0.998 [0.982; 1.015]	0.8544
	Strontium ranelate		**0.978 [0.957; 1.000]**	**0.0451**
	SERM		**0.909 [0.882; 0.938]**	**< 0.0001**
Paraclinical investigations	Monthly bisphosphonates		**2.158 [1.086; 4.288]**	**0.0281**
	Strontium ranelate		1.451 [0.608; 3.463]	0.4017
	SERM		0.495 [0.213; 1.152]	0.1028
Osteoporotic fractures	Monthly bisphosphonates		0.851 [0.632; 1.144]	0.2851
	Strontium ranelate		**1.631 [1.104; 2.411]**	**0.0141**
	SERM		**0.501 [0.300; 0.837]**	**0.0083**
Physician speciality	Monthly bisphosphonates	Rheumatologists	1.00	.
		Gynaecologists	0.908 [0.510; 1.618]	0.7431
		GPs	**1.475 [1.051; 2.071]**	**0.0247**
	Strontium ranelate	Rheumatologists	1.00	.
		Gynaecologists	**2.653 [1.486; 4.734]**	**0.0010**
		GPs	**0.605 [0.396; 0.924]**	**0.0020**
	SERM	Rheumatologists	1.00	.
		Gynaecologists	**2.153 [1.081; 4.289]**	**0.0292**
		GPs	0.857 [0.482; 1.524]	0.5998
Ongoing treatment at the time of consultation	Monthly bisphosphonates		**0.573 [0.426; 0.771]**	**0.0002**
	Strontium ranelate		1.107 [0.751; 1.633]	0.6063
	SERM		0.928 [0.577; 1.492]	0.7576

Odds ratios are presented with their 95% confidence intervals. The reference treatment group for calculation of odds ratios was women treated with weekly bisphosphonates (BP).

### Quality of life

SF-12 scores in the patient questionnaire population (N = 1,217) are presented in Table 6. Physical component scores were lowest (worse quality of life) in women consulting rheumatologists, in women complaining of pain, in women with recent fractures and in those with fractures of the spine or hip compared to other sites. Mental component scores were lower in women complaining of pain, in women with a history of fractures and in those with fractures of the spine or hip compared to other sites.

**Table 6:** SF-12 quality of life scores in the patient questionnaire population

			PCS score	MCS score
	N =	Missing data	Mean ± SD	Mean ± SD
**By fracture history**				
Fractures < 6 months	**N = 526**	12	42.74 ± 7.55	43.14 ± 10.43
Fractures >6 months	**N = 63**	3	43.51 ± 8.14	43.89 ± 11.63
No osteoporotic fracture	**N = 575**	12	48.20 ± 7.48	45.48 ± 9.82
TOTAL	**N = 1,217**	28	45.53 ± 7.97	44.35 ± 10.24
			*p ≤ 0.001*	*p = 0.001*
**By fracture site (in fractures < 6 months)**				
Spine	**N = 200**	4	40.70 ± 7.10	41.90 ± 10.47
Wrist	**N = 185**	5	45.58 ± 7.12	44.74 ± 9.98
Upper arm	**N = 17**	1	46.99 ± 8.00	45.45 ± 9.68
Hip	**N = 35**	0	40.77 ± 7.10	43.21 ± 10.76
Others	**N = 85**	2	41.03 ± 7.23	42.13 ± 11.04
			*p ≤ 0.001*	*p = 0.079*
**By presence of pain**				
Pain	**N = 647**	14	42.48 ± 7.14	42.57 ± 10.39
No pain	**N = 555**	14	49.15 ± 7.29	46.47 ± 9.54
			*p ≤ 0.001*	*p ≤ 0.001*
**By physician speciality**				
GPs	**N = 784**	94	45.22 ± 7.63	44.73 ± 10.07
Rheumatologists	**N = 276**	14	44.06 ± 8.42	43.83 ± 10.48
Gynaecologists	**N = 157**	5	49.69 ± 7.57	43.30 ± 10.62
TOTAL	**N = 1217**	28	45.53 ± 7.97	44.35 ± 10.24
			*p ≤ 0.001*	*p = 0.223*

PCS: physical component summary; MCS: mental component summary. Between group differences were determined with the Kruskal-Wallis test.

## Discussion

The principal finding of this study was that the majority of women in the study who had received a diagnosis and treatment for osteoporosis had undergone bone mass densitometry in the previous six months (85.9%). A high proportion was observed both for women followed in primary care by their general practitioner (80%) and for those followed by a specialist (94%). This figure can be compared to that of 63% for the proportion of women receiving a diagnosis of osteoporosis who had ever undergone densitometry observed in a previous survey that we performed using a very similar methodology in a primary care context in France in 2005 [10], before the introduction of the present HAS guidelines on densitometry and reimbursement. The respective figures for the proportion of non-fractured women who had undergone densitometry were 61.1% in the present study and 39.4% in the previous study. These encouraging observations suggest that the new French guidelines have already, two years after their introduction, had an impact on the uptake of densitometry in routine care in France.

Certain differences were observed in patient characteristics between the women followed in primary care and those consulting specialists. Women consulting gynaecologists were younger and had less frequently experienced fractures than those consulting rheumatologists or general practitioners. This difference in fracture rate is likely to be a consequence of the age difference. Women with multiple fracture risk factors more frequently consulted rheumatologists.

With regard to treatment, specific anti-osteoporosis treatments (bisphosphonates, SERMs or strontium ranelate) were prescribed in over ninety percent of women, with bisphosphonates being the most frequently prescribed class. Of the available bisphosphonate treatment regimens, weekly and monthly regimens were used in approximately similar proportions, with very little use of daily regimens. This is appropriate, since superior treatment compliance can be achieved using episodic compared to daily dosing regimens [14-16]. Differences in prescribing patterns were observed between the three physician groups in the multivariate analysis, with gynaecologists being more likely to prescribe SERMs than rheumatologists. This may relate to the mechanism of action of this class of medication (oestrogen receptor agonist or antagonist according to the tissue type), which may be more attractive for gynaecologists, who are likely to be particularly interested in selective endocrine regulation of bone tissue by sex hormones. General practitioners were less likely to prescribe strontium ranelate than rheumatologists, which may reflect its rather recent introduction onto the French market. On the other hand, they were more likely to prescribe monthly bisphosphonates. The observation that prescribing rates were similar between general practitioners and rheumatologists is also encouraging and suggests that national prescribing recommendations for anti-osteoporotic therapies have been rapidly disseminated and become the norm in primary care. In our previous study, GPs were already prescribing bisphosphonates to 79.8% of their patients [10], and this proportion has not changed, although there has been a shift away from daily treatments towards monthly treatments.

Apart from physician speciality, other variables associated with prescription choice were age, previous fracture history, previous paraclinical observations and whether the treatment was initiated prior to the study or following participation in the study. Strontium ranelate and SERMs were more likely to be used in younger women, whereas SERMs were less likely to be prescribed to women who had experienced a previous osteoporotic fracture. The association with age may be related to the fact that most of the evidence for fracture prevention with SERMs has come from trials performed in younger women, although it should be noted that the average age of participants in the positive MORE study in vertebral fracture was 67 years [17]. Similarly, most of the evidence for the efficacy of strontium ranelate has been obtained in younger women (aged between 50 and 65 years) [18], although both the SOTI [19] and TROPOS [20] trials showed efficacy in older women. The association with previous fracture may reflect the fact that SERMs do not have strong antiresorptive properties and may be perceived as more appropriate for preventing demineralisation in patients with no fractures, rather for restoring bone mass when a critical fracture threshold has been reached. Strontium ranelate was prescribed more frequently in patients with previous fractures, perhaps reflecting a perception of superior efficacy in restoring bone mass. The independent association between treatment choice and prescription of paraclinical investigations was unexpected, but may indicate greater use of monthly bisphosphonates in patients whose osteoporosis was difficult to manage. The association between greater monthly bisphosphonate use in women who were prescribed treatment following rather than prior to physician participation in the trial may indicate a participation bias.

With respect to quality of life, low PCS and MCS scores on the SF-12 were observed (population norm: 50). This is consistent with our previous observations using this instrument in women with osteoporosis [10]. We found that quality of life was poorer in women with recent fractures than in unfractured women, and more surprisingly, in women who had experienced fractures over six months previously. This suggests that occurrence of a fracture leads to a long-lasting decrease in quality of life. Another observation was that both components of quality of life were lowest in women who had experienced vertebral fractures compared to other fracture sites. Both components were unsurprisingly lower in women who reported experiencing pain. Women consulting rheumatologists reported lower physical quality of life than those consulting other specialities. This may be explained that by the fact that rheumatologists would be expected to see the more severe patients.

Some comments on the design of the study may be helpful. The objective was to collect information on the characteristics of postmenopausal women treated for osteoporosis in France. Reliable and exhaustive information could not be gathered by interviewing women in the general population or from prescription claims databases and it was therefore necessary to collect the information from treating physicians. In this context, it becomes important to ensure that the data collected is as representative as possible of all postmenopausal women treated for osteoporosis in France. We took several steps to optimise the representativity of the data. With respect to physicians, participants were firstly selected at random from an exhaustive national physician listing and secondly stratified by physician specialty to reflect the proportion of women who receive a diagnosis of osteoporosis from different types of physician in France, determined in a recent general population survey [9]. However, since participation was voluntary, it is possible that participants may differ from physicians who decline to participate, and indeed some small differences in demographics between participants and non-participants were observed. It is not possible to know if these two groups differ in their treatment practice and, if so, to what extent this influences the data collected. This limitation is common to all pharmacoepidemiological studies in which data is actively supplied by physicians. With respect to patients, we attempted to ensure representativity by the use of a registry. Participating physicians were requested to enter all women consulting during the study period who had undergone densitometry or experienced a fracture in the previous six months into a registry and provide a minimum amount of demographic and clinical information on these women. Participation in the registry was nonetheless capped at ten women in order to avoid centre effects caused by certain physicians contributing disproportionate numbers of patients to the study. The physicians were asked to enter the first three women consulting into the study itself. It was thus possible to compare the women included in the study with the other women in the registry to ensure that they were representative of all eligible women seen in the practice. When this was done, it was observed that disproportionately more women who had undergone densitometry were being included in the study compared to all eligible women in the registry (85.5% *versus *81.3%), opening the door to potential bias. It is possible that this arose as a result of a misunderstanding of the protocol by certain participants who entered only women who had undergone densitometry and not those with fractures into the study. In light of this difference, we performed a *post hoc *analysis to compare women included in the study on the basis of densitometry to those included on the basis of a fracture. The principal difference observed was a younger age in the former group, which is not unexpected. The potential impact of this bias on the representativity of the data is likely to be modest. For the rate of use of densitometry, this can in case be measured using all eligible patients in the registry as the denominator, which yields a rate of 81.3%. For the determinants of treatment choice, the origin of the diagnosis (densitometry or fracture) was entered into the multivariate analysis but not retained as an independent variable associated with treatment. For the other study variables, some bias in the data may exist due to slight under-representation of the women diagnosed due to a fracture, but the comparison of the two populations provided in Table 1 allows such bias to be put in perspective. The principal difference between the two groups was that women who had undergone densitometry were younger than those experiencing fractures. The other differences observed between the two groups can be accounted for by this difference in age (time since menopause, number of fracture risk factors and proportion treated with SERMs).

The study has a number of strengths and weaknesses. Amongst the strengths are the large study sample recruited from three care contexts, allowing differences between physician specialities to be assessed, the use of a similar design to a previous study, thus allowing data to be compared longitudinally, especially with respect to the impact of the new French osteoporosis management guidelines, and the range of the data collected. In addition, the comparison between physician groups is an original feature and reveals pertinent differences in patient care. Concerning the limitations of the study, the community-based design excluded *de facto *institutionalised patients, who represent an important reservoir of post-menopausal women with risk factors for osteoporotic fracture. A flaw in the implementation of the study related to treatment choice. According to the protocol, participating physicians were supposed to have already selected, and whenever possible, implemented their treatment choice when they started the study. However, in practice, treatment was initiated at the time of the reference consultation in 40% of the women. Participation in the study may thus have influenced the choice of treatment, particularly since the study sponsor markets one of the possible choices (monthly ibandronate). For this reason, the descriptive analysis of treatments presented in Table 3 has been restricted to treatments already initiated prior to the study. It should also be recognised that it may not be possible to generalise the findings obtained to other healthcare systems where post-menopausal osteoporosis may be managed differently to France. Finally, interpretation of the data on quality of life is limited by the absence of a healthy comparator group in this study.

## Conclusions

This study indicates that since the introduction of new guidelines for bone mass densitometry and reimbursement for women at risk for osteoporotic fracture, the use of densitometry has increased, with four out of five women receiving a diagnosis of osteoporosis due to low bone mass density. This relatively high rate is observed for all physician specialties involved in the diagnosis of osteoporosis. However, the type of osteoporosis patients seen and the type of antiresorptive treatment offered differs between physician specialities.

## Competing interests

AFG and FEC are employees of Laboratoires GlaxoSmithKline (GSK), France who markets ibandronate and denosumab, two anti-osteoporosis treatments. BC has received consultancy fees from GSK, Laboratoires Roche, Amgen, Novartis and Merck, Sharpe & Dohme with respect to his participation in this and other projects concerning the treatment of osteoporosis. FB, FD and DH have received consultancy fees from GSK and Laboratoire Roche with respect to their participation in this and other projects concerning osteoporosis. FM, CR and VB received stipends from GSK for data analysis (FM) or for coordination of the study (CR and VB).

## Authors' contributions

BC, FB, FD and DH made up the academic steering committee of the study. They advised on the study design, contributed to the analysis of the data and interpretation of the results, recommended the publication policy to follow and contributed to writing and critical revision of the manuscript. FM performed the data management and analysis for the study. CR and VB were responsible for the day-to-day operational management of the study. AFG and FEC conceived the study, recruited the steering committee, oversaw the implementation of the study, and initiated the preparation of the present manuscript. And finally, all authors read and approved the final manuscript.

## Pre-publication history

The pre-publication history for this paper can be accessed here:

http://www.biomedcentral.com/1471-2474/12/7/prepub

## References

[B1] Johnell O Kanis J Epidemiology of osteoporotic fractures Osteoporos Int 2005 16 Suppl 2 S3 7 10.1007/s00198-004-1702-6 15365697

[B2] Delmas PD The use of bisphosphonates in the treatment of osteoporosis Current opinion in rheumatology 2005 17 462 466 15956844 10.1097/01.bor.0000163448.51661.87

[B3] Briançon D de Gaudemar JB Forestier R Management of osteoporosis in women with peripheral osteoporotic fractures after 50 years of age: a study of practices Joint Bone Spine 2004 71 128 130 15050196 10.1016/S1297-319X(03)00060-5

[B4] Elliot-Gibson V Bogoch ER Jamal SA Beaton DE Practice patterns in the diagnosis and treatment of osteoporosis after a fragility fracture: a systematic review Osteoporos Int 2004 15 767 778 10.1007/s00198-004-1675-5 15258724

[B5] Sahota O Worley A Hosking DJ An audit of current clinical practice in the management of osteoporosis in Nottingham Journal of public health medicine 2000 22 466 472 10.1093/pubmed/22.4.466 11192273

[B6] Delmas PD van de Langerijt L Watts NB Eastell R Genant H Grauer A Cahall DL Underdiagnosis of vertebral fractures is a worldwide problem: the IMPACT study J Bone Miner Res 2005 20 557 563 10.1359/JBMR.041214 15765173

[B7] Physician's guide to prevention and treatment of osteoporosis http://www.geios.es/download.php?path=pdfs&filename=GEIOS_20090422154918_guia25.pdf

[B8] Ostéodensitométrie [absorbimétrie osseuse] sur 2 sites par méthode biphotonique http://www.has-sante.fr/portail/upload/docs/application/pdf/osteodensitometrie_rapport.pdf

[B9] Lespessailles E Cotte FE Roux C Fardellone P Mercier F Gaudin AF Prevalence and features of osteoporosis in the French general population: the Instant study Joint Bone Spine 2009 76 394 400 10.1016/j.jbspin.2008.10.008 19297229

[B10] Blotman F Cortet B Hilliquin P Avouac B Allaert FA Pouchain D Gaudin AF Cotte FE El Hasnaoui A Characterisation of patients with postmenopausal osteoporosis in French primary healthcare Drugs & aging 2007 24 603 614 10.2165/00002512-200724070-00007 17658910

[B11] Huas D Debiais F Blotman F Cortet B Mercier F Rousseaux C Berger V Gaudin AF Cotte FE Compliance and treatment satisfaction of post menopausal women treated for osteoporosis. Compliance with osteoporosis treatment BMC women's health 10 26 10.1186/1472-6874-10-26 PMC2941476 20727140

[B12] Morisky DE Green LW Levine DM Concurrent and predictive validity of a self-reported measure of medication adherence Medical care 1986 24 67 74 10.1097/00005650-198601000-00007 3945130

[B13] Ware J Jr Kosinski M Keller SD A 12-Item Short-Form Health Survey: construction of scales and preliminary tests of reliability and validity Medical care 1996 34 220 233 10.1097/00005650-199603000-00003 8628042

[B14] Cramer JA Amonkar MM Hebborn A Altman R Compliance and persistence with bisphosphonate dosing regimens among women with postmenopausal osteoporosis Current medical research and opinion 2005 21 1453 1460 10.1185/030079905X61875 16197664

[B15] Cotté FE Fardellone P Mercier F Gaudin AF Roux C Adherence to monthly and weekly oral bisphosphonates in women with osteoporosis Osteoporos Int 2010 21 1 145 155 19459025 10.1007/s00198-009-0930-1 PMC2788149

[B16] Emkey RD Ettinger M Improving compliance and persistence with bisphosphonate therapy for osteoporosis The American journal of medicine 2006 119 S18 24 10.1016/j.amjmed.2005.12.019 16563937

[B17] Barrett-Connor E Grady D Sashegyi A Anderson PW Cox DA Hoszowski K Rautaharju P Harper KD Raloxifene and cardiovascular events in osteoporotic postmenopausal women: four-year results from the MORE (Multiple Outcomes of Raloxifene Evaluation) randomized trial Jama 2002 287 847 857 10.1001/jama.287.7.847 11851576

[B18] Roux C Fechtenbaum J Kolta S Isaia G Andia JB Devogelaer JP Strontium ranelate reduces the risk of vertebral fracture in young postmenopausal women with severe osteoporosis Annals of the rheumatic diseases 2008 67 1736 1738 10.1136/ard.2008.094516 18713788 PMC2582331

[B19] Meunier PJ Roux C Seeman E Ortolani S Badurski JE Spector TD Cannata J Balogh A Lemmel EM Pors-Nielsen S The effects of strontium ranelate on the risk of vertebral fracture in women with postmenopausal osteoporosis The New England journal of medicine 2004 350 459 468 10.1056/NEJMoa022436 14749454

[B20] Reginster JY Seeman E De Vernejoul MC Adami S Compston J Phenekos C Devogelaer JP Curiel MD Sawicki A Goemaere S Strontium ranelate reduces the risk of nonvertebral fractures in postmenopausal women with osteoporosis: Treatment of Peripheral Osteoporosis (TROPOS) study The Journal of clinical endocrinology and metabolism 2005 90 2816 2822 10.1210/jc.2004-1774 15728210

